# Estimation of Plasma Volume by Machine Learning to Improve the Interpretation of the Athlete Biological Passport

**DOI:** 10.1002/dta.3938

**Published:** 2025-08-09

**Authors:** Bastien Krumm, Laura Lewis, Jakob Mørkeberg, Yorck Olaf Schumacher, Giuseppe d'Onofrio, Basile Moreillon, Raphael Faiss

**Affiliations:** ^1^ Research and Expertise in Antidoping Sciences, Institute of Sport Sciences University of Lausanne Lausanne Switzerland; ^2^ US Anti‐Doping Agency Colorado Springs Colorado USA; ^3^ Science and Research Anti Doping Denmark Brøndby Denmark; ^4^ Aspetar Orthopedic and Sports Medicine Hospital Doha Qatar; ^5^ Catholic University of the Sacred Heart Rome Italy

**Keywords:** athlete biological passport, machine learning, plasma volume

## Abstract

The identification of confounding factors related to plasma volume (PV) fluctuations is crucial for appropriate qualitative interpretations of Athlete Biological Passport (ABP) profiles. As part of ongoing efforts to remove PV variance from the concentration‐based biomarkers such as hemoglobin concentration ([Hb]), a new machine learning model for blood volume (BV) estimation using a single complete blood count analysis was applied within the ABP framework. Forty existing ABP profiles from elite athletes and healthy control subjects were used. PV was estimated using a machine learning model trained on a previous dataset. First, a visual display of the estimated PV shift was added in overlay of individual profiles. Alternatively, individual [Hb] thresholds were adjusted in a new graphical profile to account for PV variations. Finally, a set of ABP profiles with PV estimations was presented to ABP experts to assess the model's relevance in interpreting hematological data. A moderate correlation was found between measured and estimated PV in both men (*r* = 0.40, *p* < 0.0001) and women (*r* = 0.39, *p* < 0.0001), supporting the validity of the estimation model. In addition, ABP experts favorably assessed the available PV information, particularly the visual representation of PV. This novel estimation model offers distinct advantages (e.g., same biomarkers currently analyzed from routine ABP analyses) and could therefore be of particular interest. Further application of this model in the presence of specific and transient confounding factors may allow to confirm these results.

## Graphical abstract

A new machine learning model for plasma volume (PV) estimation was applied within the ABP framework. PV estimation was applied through visual displays or corrected ABP thresholds. With a favorable assessment from ABP experts, these results support further application of this model in the presence of specific confounding factors.

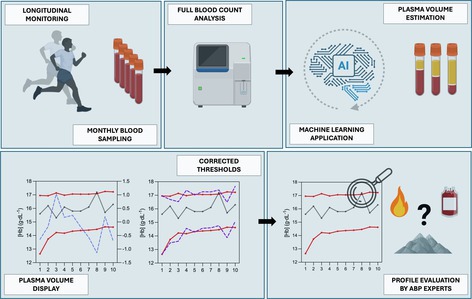

## 1 Introduction

The Athlete Biological Passport (ABP) provides individual monitoring of biomarkers to identify nonphysiological variations induced by doping substances or methods [1]. Since its application, the ABP has demonstrated indisputable positive effects [2], probably thanks to a combined effect of sensitivity and deterrence [3]. Nevertheless, ABP biomarkers may be altered by various confounding factors that possibly confuse the qualitative interpretation of individual profiles, such as plasma volume (PV) fluctuations [4]. The renin–angiotensin–aldosterone system primarily regulates PV [5], whereby diverse physiological stressors may elicit fluid redistribution between the intravascular and interstitial compartments. This results in acute PV modifications of up to 25% within a few hours, significantly changing concentration‐dependent variables [6, 7]. In this context, longitudinal monitoring of hemoglobin concentration ([Hb]) as included in the ABP may be particularly critical. Consequently, potential misinterpretation of ABP profiles may arise from deliberate hemodilution, such as acute hyperhydration [8] intended to mask abnormally elevated [Hb], or physiological changes, such as hemoconcentration due to acute exercise [9], hemodilution due to chronic exercise, or other environmental changes such as heat—or altitude exposure [10]. Given the increasing exposure of athletes to environmental stressors [11], accurate interpretation of ABP hematological profiles requires comprehensive expertise in human physiology and the various determinants of PV variability.

In this context, a preliminary approach seeking to quantify PV fluctuations for antidoping purposes was investigated by using the carbon‐monoxide (CO)‐rebreathing method [12, 13]. While the toxicity of the gas required for this procedure presents a major barrier toward its antidoping application [14], indirect biomarkers were alternatively proposed to identify PV shifts. Initial approaches focused on a single variable, such as serum albumin [7, 15], and later combined with additional serum biomarkers [16]. More recently, a multiparametric model was developed to account for PV fluctuations in the interpretation of ABP profiles [17]. Based on eight volume‐sensitive serum biomarkers [16], this model corrects adaptive thresholds for [Hb] and OFF‐score (OFFs) (i.e., the two primary parameters within the ABP hematological module [18]). The OFFs is a reliable indirect indicator of blood doping [1] calculated using the formula [Hb] × 10–60 × √reticulocytes percentage (RET%). Representing a major step forward in the evolution of the ABP, this multiparametric PV model proved to be effective in interpreting transient PV variations such as consecutive racing days [19] or 3 weeks of simulated *live‐high train‐low* altitude training camp [20]. Nevertheless, this model did not consistently enhance the interpretation of blood profile variations in elite cross‐country skiers monitored over one year [21]. In addition, the inherent necessity of additional serum analysis required for this corrective model challenges its practical applicability [15].

An alternative model for estimating blood volume (BV) based on machine learning was recently tested [22]. The model aims to estimate PV and total hemoglobin mass (Hbmass) based on data derived from a single complete blood count analysis (CBC) and anthropological data. The application of this model for antidoping purposes may offer an effective and readily implementable approach to enhance the interpretation of ABP profiles, as it leverages the same hematological variables already collected under stringent sampling and analytical protocols. This study, therefore, aims to apply a machine learning PV estimation model in the review process of ABP profiles from elite athletes and healthy control subjects. It was hypothesized that PV estimation could facilitate the assessment of ABP profiles through an easily applicable machine learning model.

## 2 Methods

Hematological profiles from 20 elite cross‐country skiers (16 men and 4 women) and 20 healthy sports sciences students (11 men and 9 women) were included. The full design for the monitoring study, allowing for the production of individual profiles, was previously described elsewhere [21]. Briefly, participants visited the laboratory monthly for one year, with each visit including a concomitant venous blood sample with CBC and duplicate BV measurements by CO‐rebreathing.

### 2.1 Hematological Analysis and ABP Profiles

The CBC variables (as ABP biomarkers) were obtained by flow cytometry (Sysmex XN‐series analyzers, Norderstedt, Germany) from a venous blood sample, following the current blood collection and analyses guidelines in force for ABP analyses [18, 22, 23]. The ABP profiles were generated with the Anti‐Doping Administration & Management System (ADAMS) training platform provided by the World Anti‐Doping Agency (WADA) as originally reported in a previous publication [21] and reproduced by using a graphing software (PRISM Version 9, GraphPad Software Inc., La Jolla, CA, USA). Hbmass and BVs were quantified through the optimized CO‐rebreathing method using a fully automated instrument (Detalo Health, Birkerød, Denmark) as fully detailed elsewhere [24].

### 2.2 Machine Learning Predictive Model for PV

The PV estimations were performed by using a machine‐learning model through the MATLAB regression learner app (MATLAB R2022b, MathWorks Inc., Natick, USA) as recently published [22]. Briefly, the predictive model was first trained using a set of 384 data points (with concomitant CBC and BV) from previous studies performed on various populations (i.e., healthy men and women, free‐divers, and elite endurance athletes). Then, based on the corresponding CBC and anthropometric values (sex, age, height, and weight), the machine‐learning model was applied to estimate individual PV at each subject's visit. Anthropometric information was included in the development of the predictive model to enhance the accuracy of absolute value estimations. However, in the antidoping context, such data and particularly body weight can be challenging to obtain reliably. Nevertheless, as the current ABP framework focuses on evaluating PV variations between samples rather than absolute values, excluding weight from the predictive model is unlikely to significantly impact the results or the interpretation of ABP profiles. Therefore, it appears feasible to retrain the machine‐learning model in the future without this parameter. Finally, a sensitivity analysis was performed to evaluate the weight of each CBC marker in the PV estimate. To this end, each marker was individually increased or decreased by ±0.2%, ±0.1%, and ±0.05%, respectively, before repeating the estimation. The differences relative to the initial PV estimated values (in mL) were then calculated.

### 2.3 Application of PV Estimation to ABP Profiles

**FIGURE 1:**
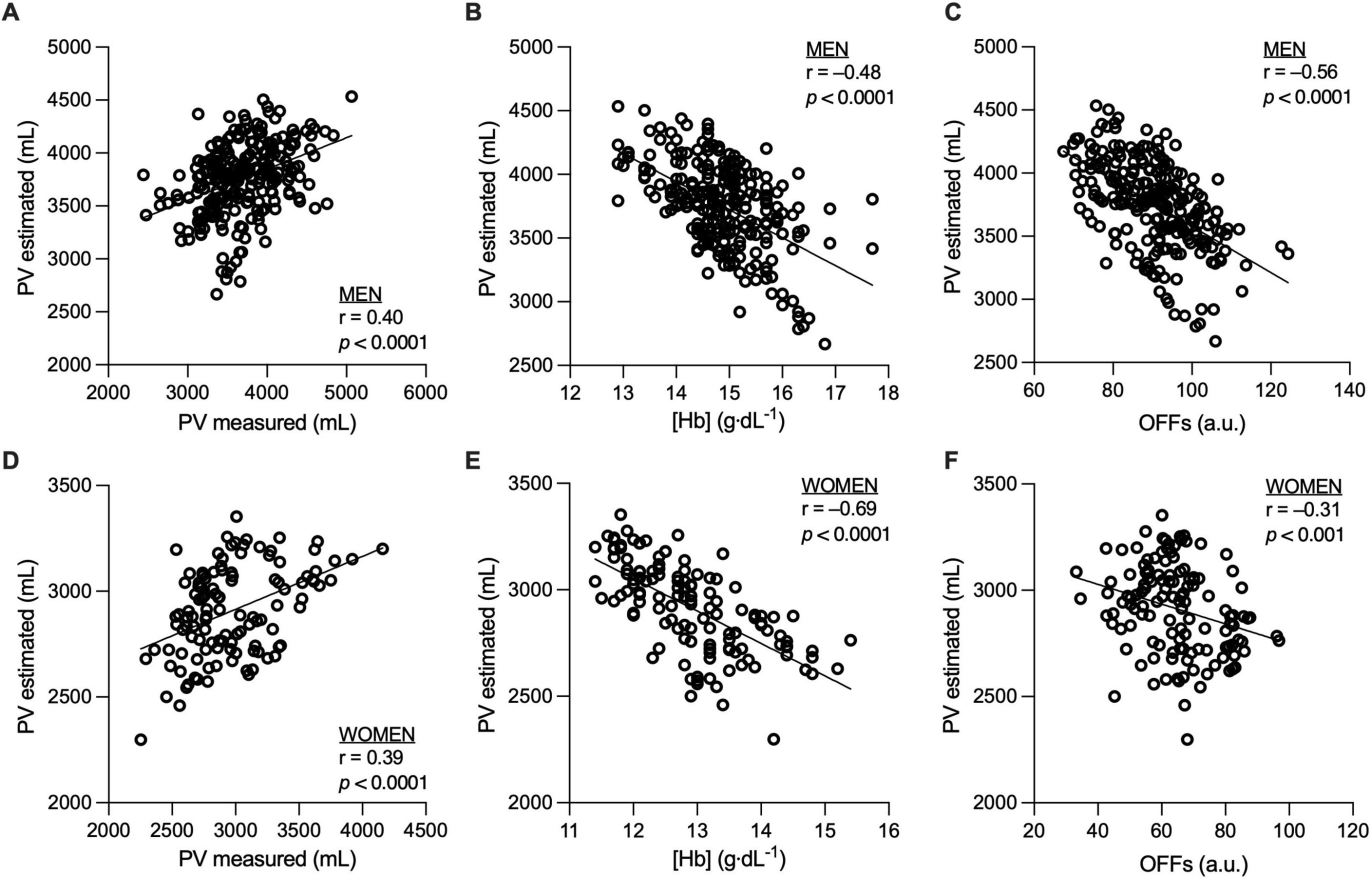
Correlation between estimated plasma volume and measured hematological variables. Correlations and simple linear regressions are reported between estimated plasma volume and measured plasma volume (via CO‐rebreathing method), hemoglobin concentration ([Hb]), and OFF‐score (OFFs) for both men (A–C) and women (D–F).

**FIGURE 2:**
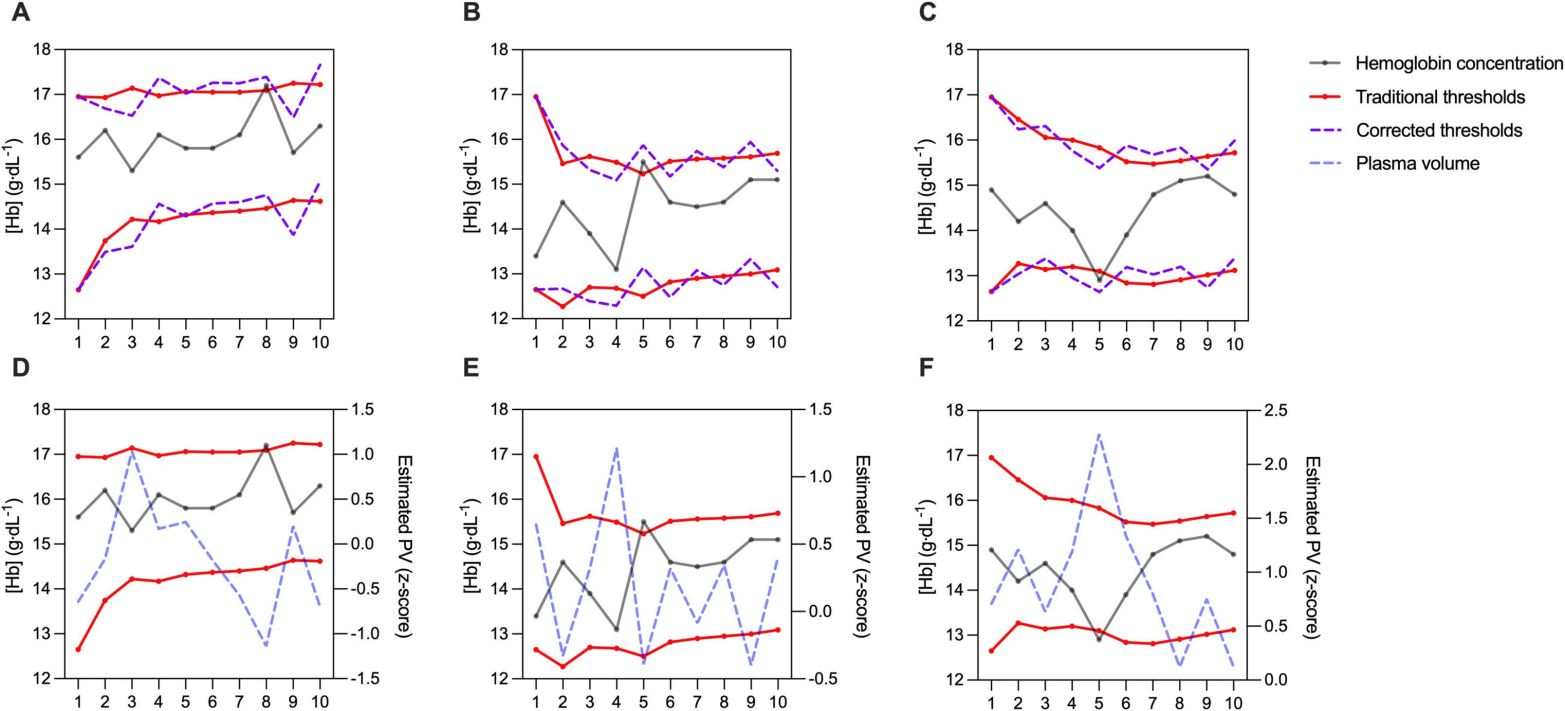
Suggestions for incorporating plasma volume into athlete biological passports (ABP) profiles. Three examples of ABP with hemoglobin concentration ([Hb]) from elite athletes are presented, each showing corrected thresholds (A–C) and plasma volume displays (D–F). The dark line represents true [Hb] values, the red lines the traditional ABP individual reference limits, the purple lines the corrected limits, and the blue line the plasma volume display expressed as a *z*‐score.

Based on PV estimation, two graphical representations were produced in addition to the original ABP graphical displays. First, a visual display of the estimated PV shift (*z*‐score) using an additional right *Y*‐axis of the individual profiles was produced in overlay of the existing profiles. Secondly, a graphical correction of individual thresholds for [Hb] was proposed. After determining the PV (in mL) for each point using the above‐mentioned machine learning model, the relative difference in PV between two successive points was computed to assess fluid shift (ΔPV_relative_). As the variability in [Hb] is only partially explained by PV variability, a weighting index was defined by measuring the determination coefficient (*R* ^2^) from the linear regression between estimated PV and [Hb], yielding values of 0.28 for men and 0.42 for women (Figure 1). In other words, we hypothesized that 28% and 42% of the variation in [Hb] could be attributed to PV fluctuations. Accordingly, these weighting factors were applied to the ΔPV_relative_ by multiplication to specifically correct the proportion of [Hb] variation attributable to PV changes (ΔPV_weighted_). The correction to be applied was finally calculated by multiplying the average of the upper and lower limits of [Hb] (in g·dL^−1^) by the ΔPV_weighted_. In this way, the original interval computed by the ABP Bayesian model remains unchanged (e.g., if the interval calculated by ADAMS between the upper and lower limit is 2.9 g·dL^−1^, as is the case for the third sample in Figure 2A, this interval remains unchanged after the correction is applied). The detailed steps and equations used to correct [Hb] thresholds are presented in Figure S1. Since OFFs is only partially influenced by PV fluctuations due to the integration of RET% (which is PV independent), the model was not adapted for this specific ABP variable.

### 2.4 Evaluations of Longitudinal Profiles by ABP Experts

To evaluate the model's relevance in the interpretation of hematological profiles, a selection of 20 ABP profiles was submitted to four independent ABP experts. To ensure interpretations as close as possible to real‐world conditions, the experts were informed that the profiles originated from various studies, with or without specific interventions (e.g., control, altitude training, heat training, treatement with recombinant erythropoietin, or blood transfusion). Nonetheless, all included subjects were presumed to be undoped. The dataset consisted of 10 profiles that triggered at least one atypical passport finding (ATPF), defined as values falling outside the individual limits for one of the two primary markers (i.e., [Hb] and OFFs), as well as 10 additional profiles. Secondary hematological variables commonly available for ABP evaluations (e.g., hematocrit or immature reticulocyte fraction) were provided to the experts. However, no additional information, such as exposure to hypoxia, heat, or time of collection, was provided.

The experts were asked to conduct a two‐step qualitative assessment: first, by evaluating the ABP profiles using standard procedures, and second, by reassessing them with additional PV information available. All responses were collected using analog scales (Figures S2 and S3), subsequently converted into numeric scales (from 1 to 10). Hence, without considering PV estimation, experts were initially asked to evaluate the complexity of interpretation (from *very easy* to *very complex*). Then, using the same evaluation criteria described in the WADA International Standard for Result Management [25], ABP profiles were rated as *normal*, *suspicious*, *likely doping*, or *likely medical condition*. Finally, based on the provided PV, experts evaluated the impact of including PV estimation windows for the corrected limits (*did the corrected limits alter your interpretation?*) and the displayed PV (*did displaying PV only alter your interpretation?*) on their evaluation (from *not at all* to *yes, a lot*).

### 2.5 Statistical Analyses

The normality of the distributions was tested with the D'Agostino and Pearson test. PV estimation and the corresponding sensitivity analyses were performed as previously described. Nonparametric Spearman correlations were performed to compare PV estimated (using the regression learner app) to PV measured (by CO‐rebreathing method), [Hb], and OFFs for both men and women. Bland–Altman analyses were conducted to assess the agreement between estimated and measured PV. Pearson correlation coefficients were performed to investigate the relationship between the experts' answers. All relationships were further examined using simple linear regressions. PV *z*‐scores used for visual representations of the displayed PV were calculated using the mean and standard deviation of each sex. The level of significance was set at *p* < 0.05. All statistical analyses and figures were generated using GraphPad PRISM Version 9 (GraphPad Software Inc., La Jolla, CA, USA).

## 3 Results

A correlation was observed between estimated and measured PV in both men (*r* = 0.40, *p* < 0.0001) and women (*r* = 0.39, *p* < 0.0001) (Figure 1). Bland–Altman analysis indicated a mean bias of 74 mL for men and −63 mL for women. Negative correlations were found between PV and [Hb] in men (*r* = −0.48, *p* < 0.0001) and women (*r* = −0.69, *p* < 0.0001), as well as between PV and OFFs in men (*r* = −0.56, *p* < 0.0001) and women (*r* = −0.31, *p* = 0.0005). When processed in ADAMS, [Hb] thresholds were exceeded at six different time points for the elite athletes' cohort and two for the healthy subjects' cohort (a total of 8 ATPFs for [Hb] only). Detailed ABP results are reported elsewhere [21]. When applying the adjusted thresholds on traditional ABP profiles, 75% (6/8) of [Hb] generated ATPFs could be explained by estimated PV fluctuations (Figure S4–S7). No additional outliers were generated.

**FIGURE 3:**
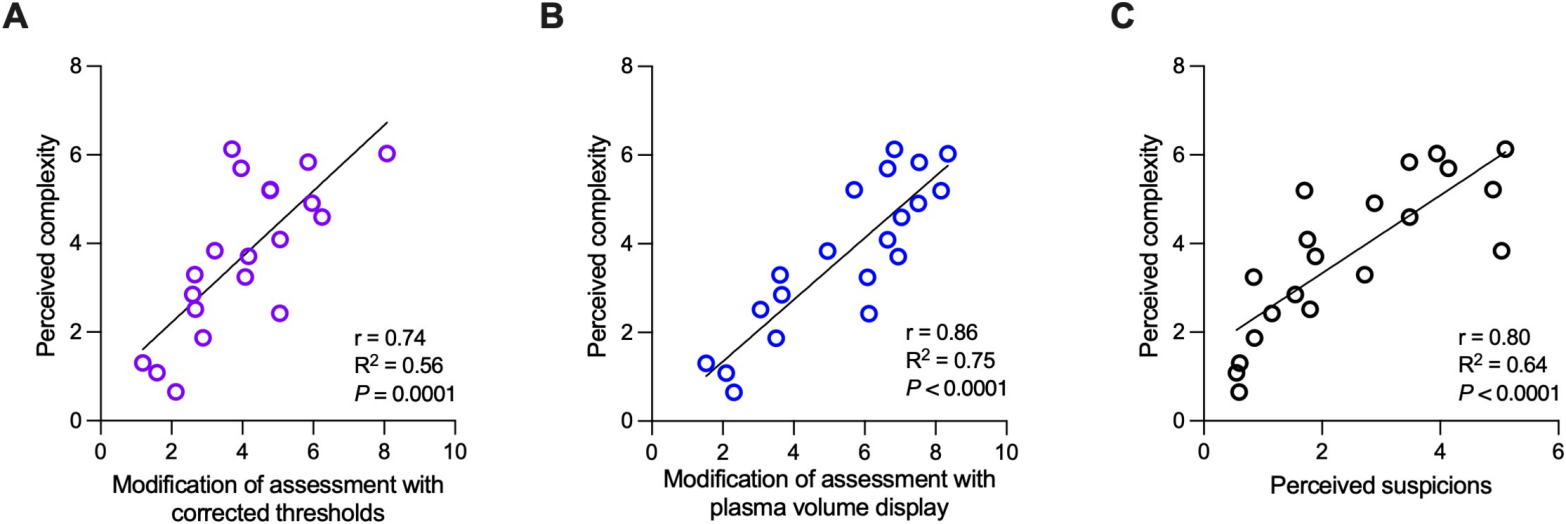
Expert evaluation of athlete biological passports profiles with and without plasma volume estimation. Correlations are reported between perceived profile complexity and changes in assessment based on corrected thresholds (A), plasma volume display (B), and perceived suspicion levels (C).

**FIGURE 4:**
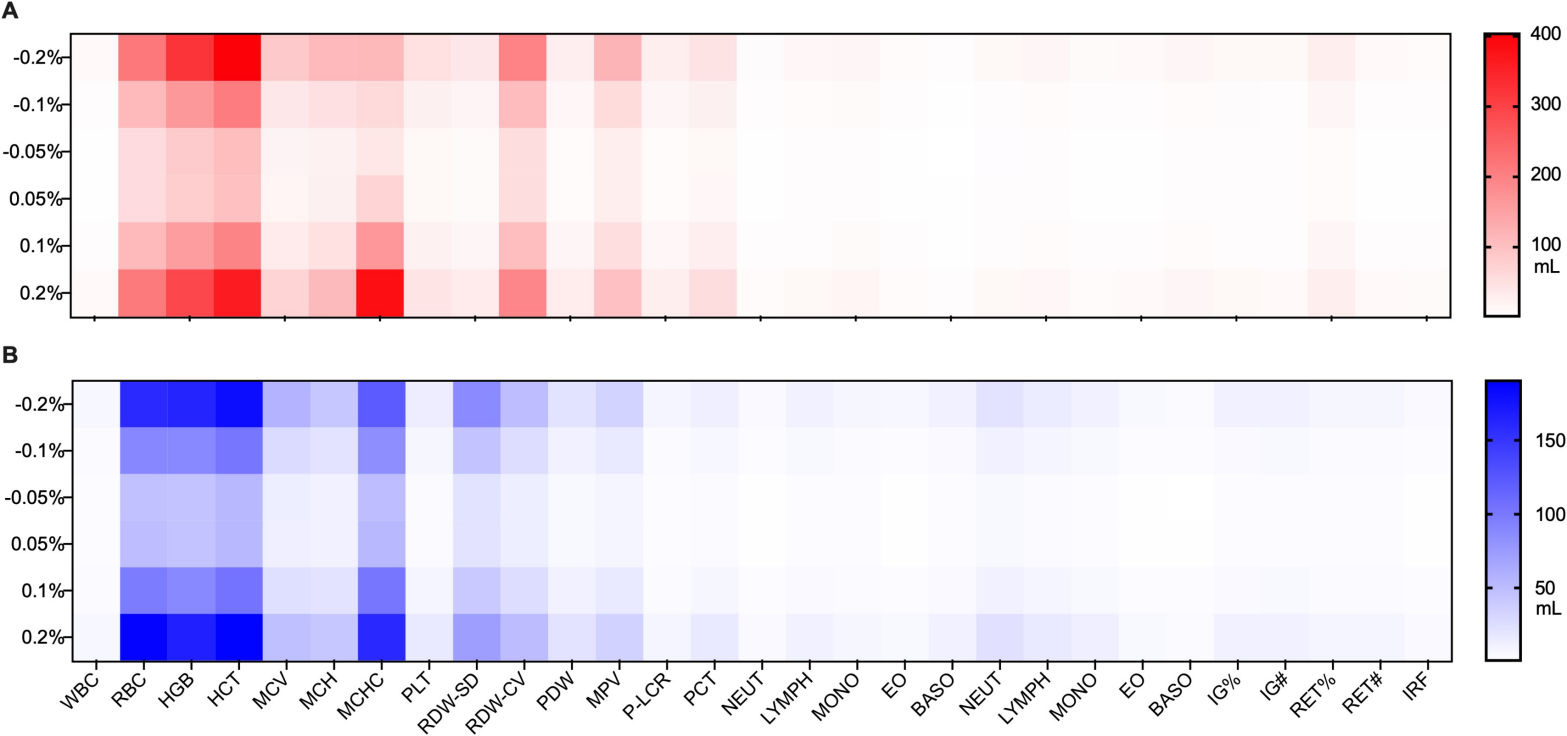
Sensitivity analysis of variables included in the machine learning predictive model. Each marker was increased or decreased by ±0.2%, ±0.1%, and ±0.05% before repeating the plasma volume estimation for men (A) and women (B). The differences to the initial estimated values (in mL) were then calculated and visualized in a heat map. A color close to white indicates low predictive power, while a color close to red indicates high predictive power. The vertical axis show percentage changes applied to each marker while the horizontal axis displays the different variables included in the model.

On a scale from 0 to 10, the complexity of ABP profiles was rated at 3.7 ± 1.7 (Figure 3). Similarly, to the question “*did displaying PV only alter your interpretation?”* experts' average score on the 1–10 scale was 5.4 ± 2.1 and 4.1 ± 1.7 to the question “*did the corrected limits alter your interpretation?”*. Perceived complexity was significantly correlated with perceived suspicion levels (*r* = 0.80, *R* ^2^ = 0.64, *p* < 0.0001). Similarly, a positive correlation was reported between profiles' complexity and changes in assessment based on corrected thresholds (*r* = 0.74, *R* ^2^ = 0.56, *p* = 0.0001) as well as PV display (*r* = 0.86, *R* ^2^ = 0.75, *p* < 0.0001), that is, the more complex the profiles were judged, the more useful the additional PV information was. Finally, the sensitivity analysis revealed that RBC, HCT, [Hb], and MCHC were the markers with most weight in PV estimations (Figure 4): ±2% change in these variables (men and women combined) resulted in additional variations of ±197, ±250, ±305, and ±205 mL, respectively.

## 4 Discussion

A newly developed machine learning model for PV estimation was applied within the ABP paradigm. Independent ABP experts favorably assessed the display of the estimated PV for each time point reported, particularly the additional visual representation of PV in overlay to existing profiles. Such a machine learning model could support an improved interpretation of volume‐sensitive biomarkers in ABP analysis by providing an alternative estimation approach.

The susceptibility of several markers included in the hematological module to PV shifts was identified as a major limitation of the ABP model from its first implementation and remains a major challenge of indirect detection [1]. Similarly, variables from other ABP modules, such as the endocrine module, are also affected by changes in PV [26]. In this context, a multiparametric approach was suggested to remove PV variance from the ABP [17]. However, despite validation in short‐term contexts [19, 20], this approach requires complementary serum analyses, and recent results highlighted some limitations when applied longitudinally [21]. Building on a recently published method for BV estimation [22], the present machine learning model may offer a complementary approach to support the longitudinal interpretation of concentration‐based biomarkers included in the ABP. Despite a weaker correlation than initially published [22], the relationship between measured PV (by CO‐rebreathing) and estimated PV remains significant for both men (*r* = 0.40, *p* < 0.0001) and women (*r* = 0.39, *p* < 0.0001) (Figure 1).

When PV estimation is applied to traditional ABP profiles (Figure 2), the adjusted thresholds are allowed to exclude six out of eight original ATPFs generated for [Hb] [21]. Since the original profiles were produced from subjects that were most probably not undergoing blood doping, it means that the originally observed ATPFs were due to physiological confounders affecting [Hb] due to PV shifts. In this context, an ideal application of the ABP framework would be to either not yield any ATPF or require experts to rule them out based on their interpretation of underpinning confounders. The removal of the majority of ATPFs with the PV estimation model supports its applicability within the ABP framework. Nevertheless, beyond the quantitative findings, the qualitative expert evaluation of ABP profiles remains essential to the ABP process. This is highlighted by the experts' evaluation of the two PV estimation displays: although both versions were favorably assessed, the displayed PV seems to have a greater influence on their interpretation compared to the corrected limits when correlated to the profile's complexity (*r* = 0.86, *R* ^2^ = 0.75, *p* < 0.0001 vs. *r* = 0.74, *R* ^2^ = 0.56, *p* = 0.0001) (Figure 3). Consequently, the aim of the present model is not to reduce the number of ATPF per se but rather to assist the experts in the interpretation of the fluctuations in [Hb]. An adjustment of individual lower and upper thresholds may therefore eventually serve as a tool to guide an expert interpretation by corroborating the visual information provided with the overlaid PV estimations.

The machine learning approach offers several key advantages. From an analytical perspective, the model requires the same biomarkers currently analyzed from routine ABP analyses [18]. With the regular increased workloads and costs for antidoping laboratories due to the expansion of targeted biomarkers (e.g., new biomarkers of the endocrine module or the blood steroidal profile) [27], the introduction of additional variables represents a further burden. Furthermore, the analytical variability needs to be considered in the development of new markers for antidoping purposes [28], especially in a longitudinal approach where samples (from the same athlete) are analyzed in different laboratories. All CBC variables used to perform PV estimation are generated from the currently used reference analyzer (the Sysmex XN‐series platform) [18].

Based on its intrinsic machine‐learning approach, it will be possible to further improve the model by training it with additional data (composed of a concomitant measurement of BVs and CBC). As BV measurements are commonly used in sports science and progressively implemented as routine monitoring in elite athletes [29], it is realistic that the model could be significantly strengthened with additional data. Therefore, with a progressively increased database with refined and diverse reference populations, the machine‐learning estimation could be further optimized to reach minimized estimation errors. Finally, the simplicity of use of the regression learner app functionality of a widely accessible software (i.e., MATLAB) is another asset for its application. After traditional ABP analysis, a simple routine could provide instant PV estimation without any additional manipulation to perform. Overall, considering the complementary properties of the current machine learning approach with the serum‐based corrective model originally suggested [17], a combined perspective might be of particular interest. For example, this model could be used if a serum sample is not available to perform the serum‐based corrective model or for confirmation in case of suspicious variations.

Omnipresent in contemporary society, machine learning and artificial intelligence have already been the focus of various antidoping research for several years [30, 31]. For example, a machine learning‐based approach was recently suggested to identify the presence of rhEPO in blood samples [32]. Nevertheless, despite obvious potential in a very fast‐growing field, these approaches are not without limitations. First, the black‐box nature of such models makes it challenging for antidoping authorities to provide transparent and scientifically justified explanations for decisions based on their outputs. Therefore, although sensitivity analyses can provide insights into the relative importance of the markers included in the prediction, quantifying the exact contribution of each variable remains challenging. Furthermore, given the limited volume of antidoping research data currently available for training compared to other fields (e.g., healthcare), the risk of overfitting necessitates careful consideration.

In this context, the suggested model is preliminary and warrants further validation, such as examining the impact of identified confounding factors (e.g., altitude or heat training) on the estimated PV to ensure the accuracy and performance of the model. Indeed, while the application of the model seems particularly well‐suited to longitudinal monitoring, the model's ability to identify abrupt PV fluctuations needs to be investigated. Finally, the sensitivity analysis identified RBC, [Hb], HCT, and MCHC as the variables with the highest weight in the resulting predictive value (Figure 4). This is not surprising, as [Hb] and HCT have been used to indirectly quantify PV shifts for decades [33]. Similarly, [Hb], in combination with serum markers, was identified as one of the eight variables incorporated into the initial multiparametric model applied to the ABP [17]. Nevertheless, this inevitably raises critical questions within the present context. Hence, due to their sensitivity to erythropoietic alterations, these markers are part of the hematological module. Therefore, to strictly avoid any dissimulation of true‐positive cases, the model's sensitivity to confirmed blood doping scenarios (e.g., blood transfusion) necessitates careful examination.

## 5 Conclusion

Consideration of confounding factors impacting PV remains critical for the interpretation of concentration‐based biomarkers. In this study, a recently developed machine learning model for estimating BV was applied to the ABP framework. A visual display of the estimated PV shift was included in the overlay of individual profiles. Alternatively, individual [Hb] thresholds were corrected in a new graphical profile to consider PV variations. The promising outcomes related to PV estimation were further supported by the favorable evaluations presented by ABP experts. By providing an alternative estimation approach, this machine learning model may complement ongoing research and facilitate ABP interpretation. Further studies, including short‐term PV variations and doping scenarios, are needed to confirm the interest of this approach.

## Conflicts of Interest

The authors declare no conflicts of interest.

## Supporting information


**Figure S1:** Illustration of the methodology applied for correcting individual hemoglobin concentration limits. The figure presents (A) a summary of all analytical steps, (B) the complete correction equations, and (C) an illustrative example.

**Figure S2:** Example of the evaluation form provided to experts for evaluating athlete biological passports (ABP) profiles with or without plasma volume estimation. The upper part includes the primary ABP markers, namely, hemoglobin concentration ([Hb]), OFF‐score (OFFs), reticulocyte percentage (RET%), and abnormal blood profile score (ABPS). The lower part was initially hidden and then shown to the expert. It includes [Hb] profiles with plasma volume estimation overlayed, where the dark line represents [Hb] values, the red lines the traditional ABP individual limits, the purple lines the corrected limits, and the gray line the plasma volume display expressed as a *z*‐score.

**Figure S3:** Example of the response form provided to experts for evaluating athlete biological passports profiles with or without plasma volume estimation. All responses were collected using visual analogic scales, subsequently converted into numeric scales.

**Figure S4:** Athlete biological passports (ABP) profiles incorporating corrected individual limits for hemoglobin concentration ([Hb]) in elite subjects (*n* = 20). The dark line represents [Hb] values, the red lines the official ABP individual limits, and the purple lines the corrected limits. The green bands represent atypical passport findings that can be explained by a shift in plasma volume.

**Figure S5:** Athlete biological passports (ABP) profiles incorporating corrected individual limits for hemoglobin concentration ([Hb]) in control subjects (*n* = 20). The dark line represents [Hb] values, the red lines the official ABP individual limits, and the purple lines the corrected limits. The green bands represent atypical passport findings that can be explained by a shift in plasma volume.

**Figure S6:** Athlete biological passports (ABP) profiles incorporating plasma volume display for hemoglobin concentration ([Hb]) in elite subjects (*n* = 20). The dark line on the left *Y*‐axis represents [Hb], with red lines indicating the official ABP individual limits. The blue line on the right *Y*‐axis shows plasma volume variation expressed as a *z*‐score.

**Figure S7:** Athlete biological passports (ABP) profiles incorporating plasma volume display for hemoglobin concentration ([Hb]) in control subjects (*n* = 20). The dark line on the left *Y*‐axis represents [Hb], with red lines indicating the official ABP individual limits. The blue line on the right *Y*‐axis shows plasma volume variation expressed as a *z*‐score.

## Data Availability

The data that support the findings of this study are available from the corresponding author upon reasonable request.
