# Supplementary figures and images for: Estimation of Plasma Volume by Machine Learning to Improve the Interpretation of the Athlete Biological Passport
Source: Drug Test Anal. 2025 Aug 9;17(11):2283–90. doi: 10.1002/dta.3938 (PMC12580168; doi:10.1002/dta.3938)

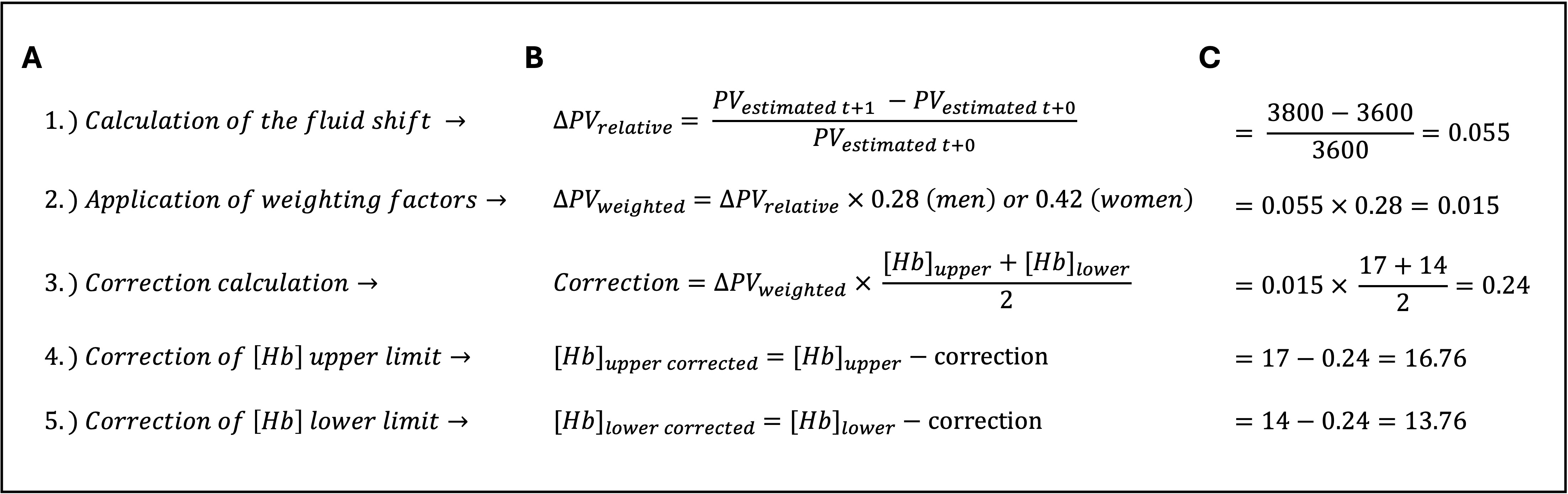

Supplement: Supplementary file 1 — Figure S1: Illustration of the methodology applied for correcting individual hemoglobin concentration limits. The figure presents (A) a summary of all analytical steps, (B) the complete correction equations, and (C) an illustrative example. [file DTA-17-2283-s003.jpg]

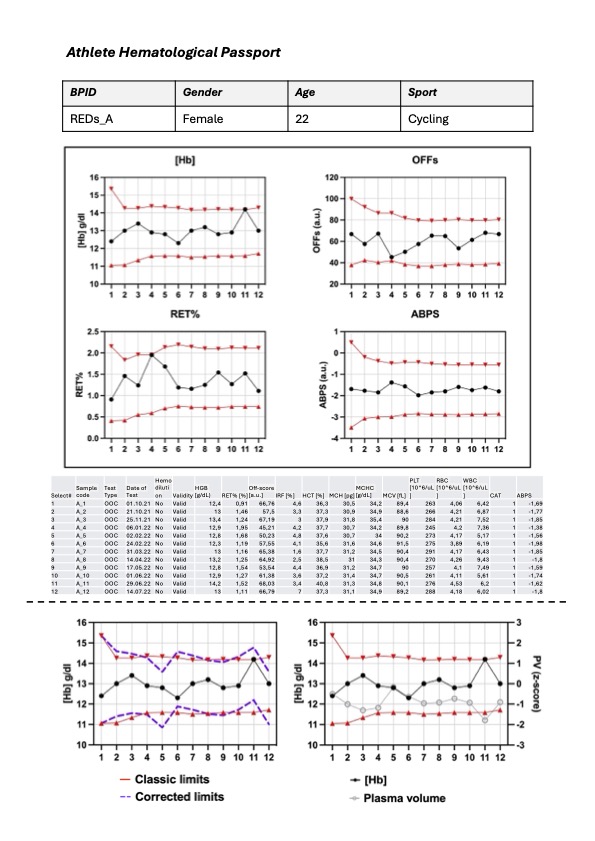

Supplement: Supplementary file 2 — Figure S2: Example of the evaluation form provided to experts for evaluating athlete biological passports (ABP) profiles with or without plasma volume estimation. The upper part includes the primary ABP markers, namely, hemoglobin concentration ([Hb]), OFF‐score (OFFs), reticulocyte percentage (RET%), and abnormal blood profile score (ABPS). The lower part was initially hidden and then shown to the expert. It includes [Hb] profiles with plasma volume estimation overlayed, where the dark line represents [Hb] values, the red lines the traditional ABP individual limits, the purple lines the corrected limits, and the gray line the plasma volume display expressed as a z‐score. [file DTA-17-2283-s001.jpg]

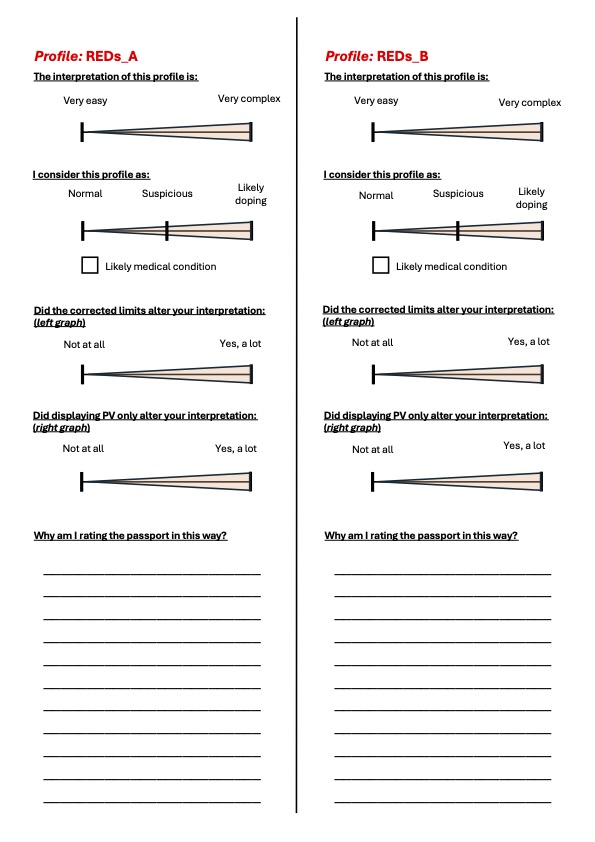

Supplement: Supplementary file 3 — Figure S3: Example of the response form provided to experts for evaluating athlete biological passports profiles with or without plasma volume estimation. All responses were collected using visual analogic scales, subsequently converted into numeric scales. [file DTA-17-2283-s004.jpg]

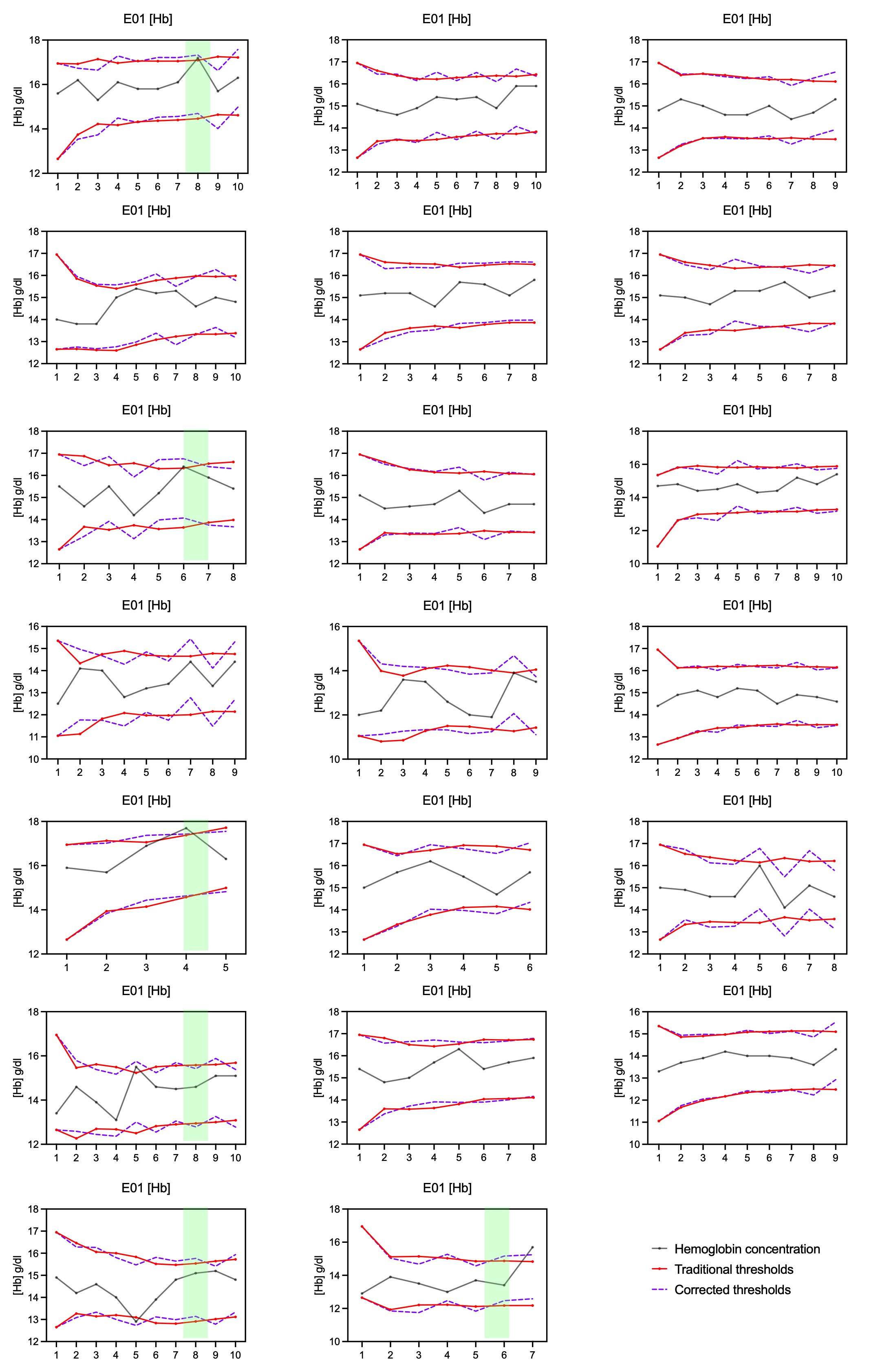

Supplement: Supplementary file 4 — Figure S4: Athlete biological passports (ABP) profiles incorporating corrected individual limits for hemoglobin concentration ([Hb]) in elite subjects (n = 20). The dark line represents [Hb] values, the red lines the official ABP individual limits, and the purple lines the corrected limits. The green bands represent atypical passport findings that can be explained by a shift in plasma volume. [file DTA-17-2283-s005.jpg]

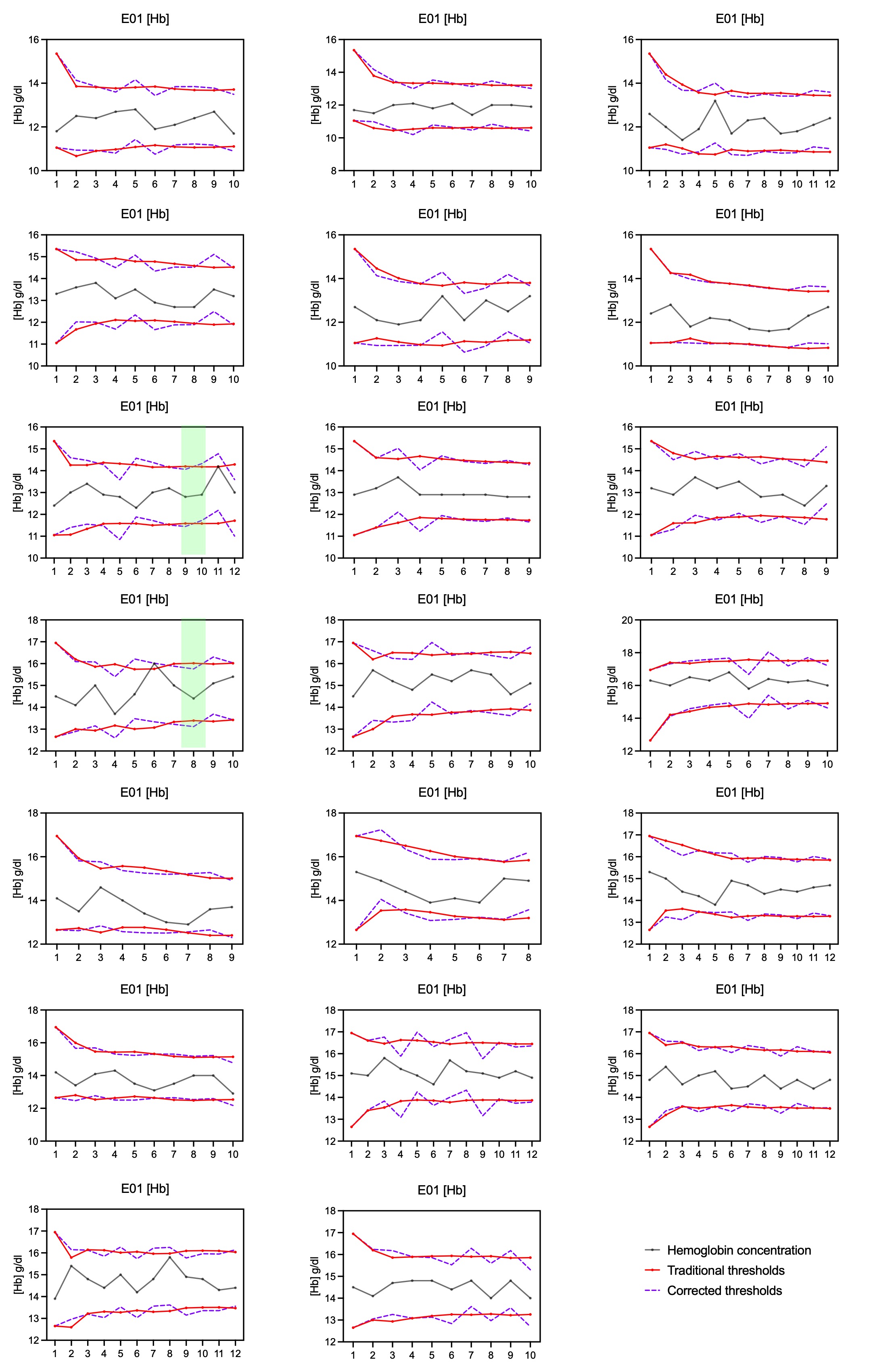

Supplement: Supplementary file 5 — Figure S5: Athlete biological passports (ABP) profiles incorporating corrected individual limits for hemoglobin concentration ([Hb]) in control subjects (n = 20). The dark line represents [Hb] values, the red lines the official ABP individual limits, and the purple lines the corrected limits. The green bands represent atypical passport findings that can be explained by a shift in plasma volume. [file DTA-17-2283-s002.jpg]

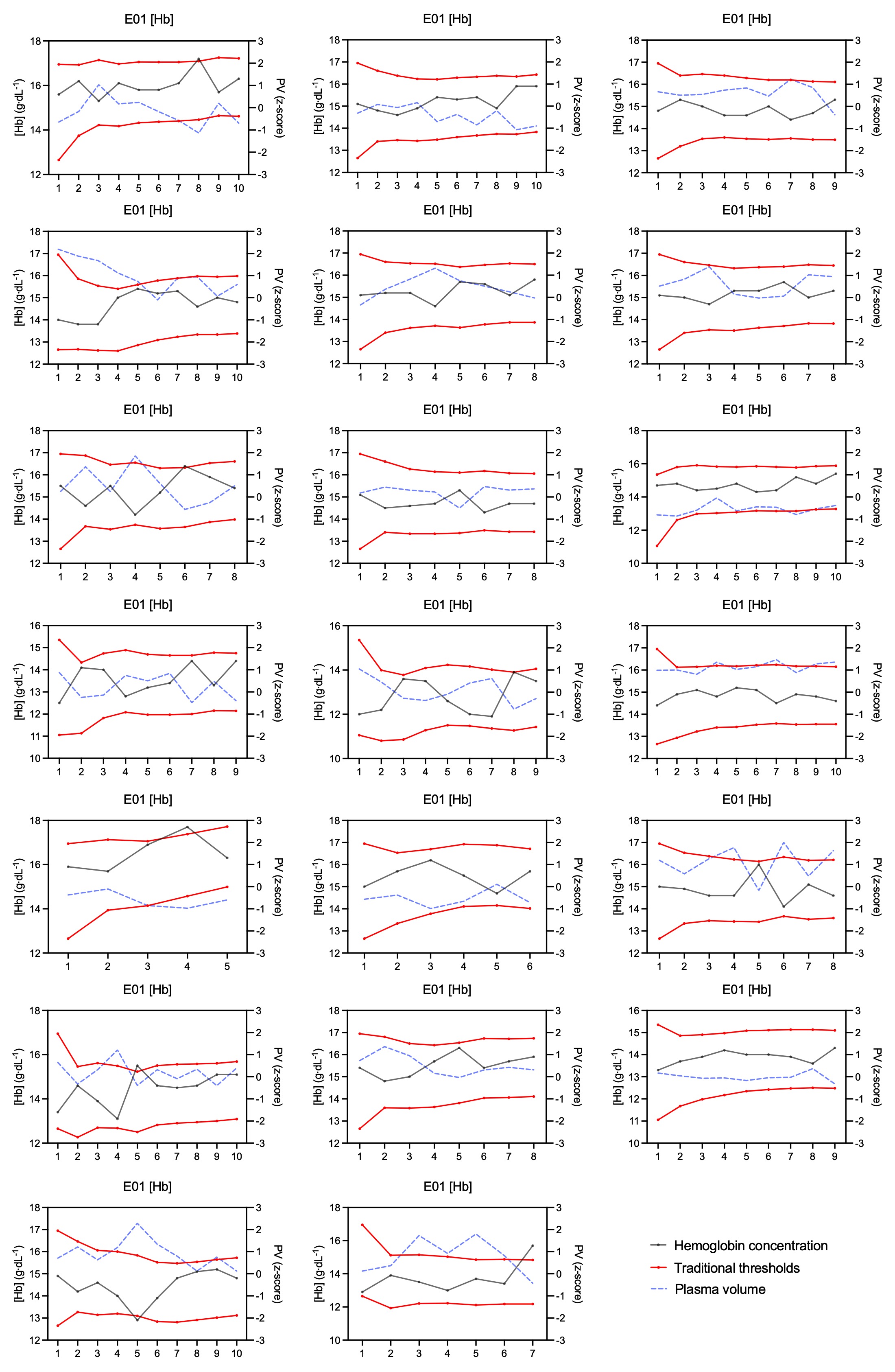

Supplement: Supplementary file 6 — Figure S6: Athlete biological passports (ABP) profiles incorporating plasma volume display for hemoglobin concentration ([Hb]) in elite subjects (n = 20). The dark line on the left Y‐axis represents [Hb], with red lines indicating the official ABP individual limits. The blue line on the right Y‐axis shows plasma volume variation expressed as a z‐score. [file DTA-17-2283-s006.jpg]

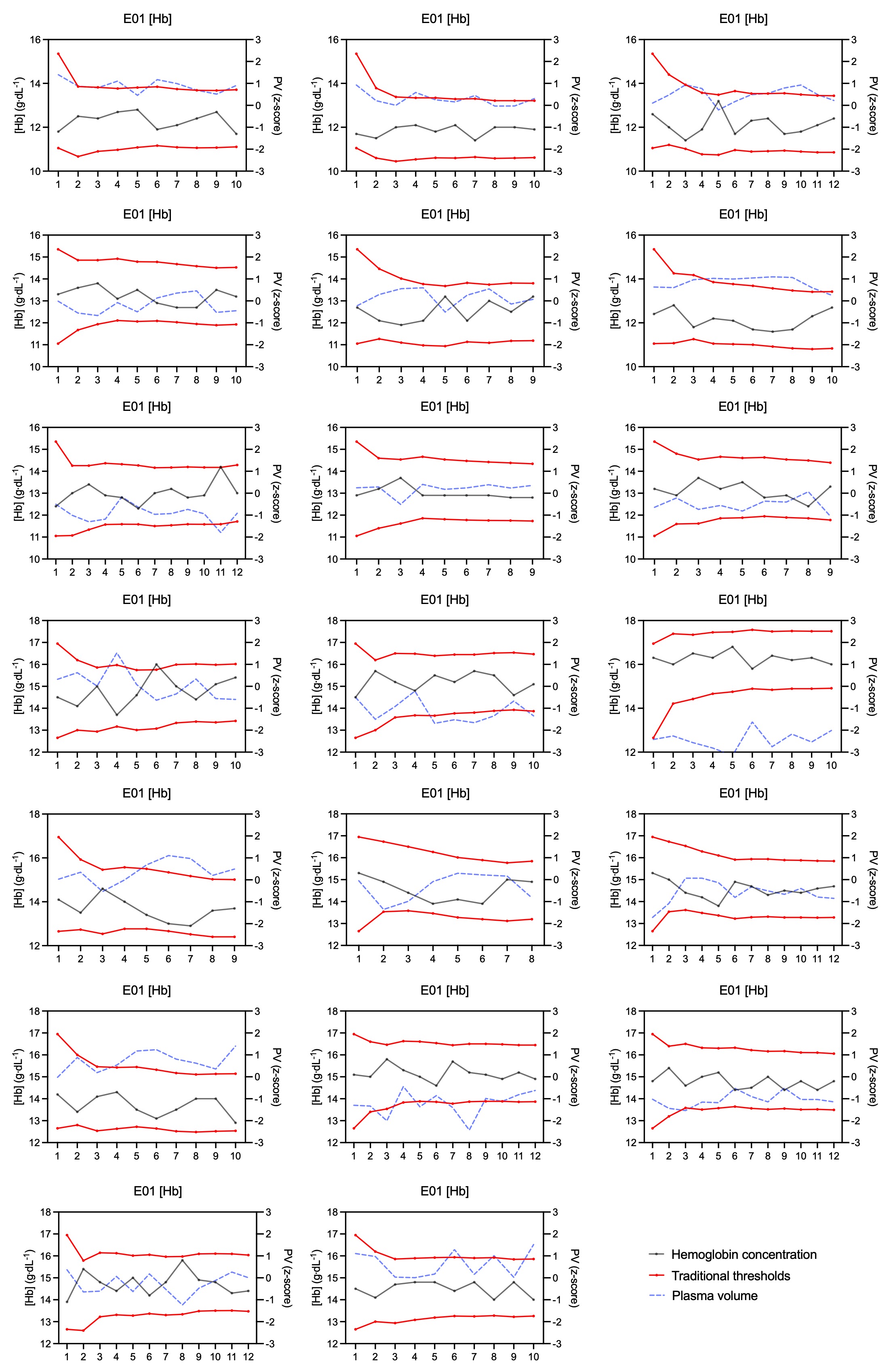

Supplement: Supplementary file 7 — Figure S7: Athlete biological passports (ABP) profiles incorporating plasma volume display for hemoglobin concentration ([Hb]) in control subjects (n = 20). The dark line on the left Y‐axis represents [Hb], with red lines indicating the official ABP individual limits. The blue line on the right Y‐axis shows plasma volume variation expressed as a z‐score. [file DTA-17-2283-s007.jpg]
